# Mosaic and Intronic Mutations in *TSC1/TSC2* Explain the Majority of TSC Patients with No Mutation Identified by Conventional Testing

**DOI:** 10.1371/journal.pgen.1005637

**Published:** 2015-11-05

**Authors:** Magdalena E. Tyburczy, Kira A. Dies, Jennifer Glass, Susana Camposano, Yvonne Chekaluk, Aaron R. Thorner, Ling Lin, Darcy Krueger, David N. Franz, Elizabeth A. Thiele, Mustafa Sahin, David J. Kwiatkowski

**Affiliations:** 1 Department of Medicine, Brigham and Women’s Hospital, Boston, Massachusetts, United States of America; 2 Department of Neurology, Boston Children’s Hospital, Boston, Massachusetts, United States of America; 3 Department of Human Genetics, Cincinnati Children’s Hospital Medical Center, Cincinnati, Ohio, United States of America; 4 Department of Neurology, Massachusetts General Hospital, Boston, Massachusetts, United States of America; 5 Center for Cancer Genome Discovery, Dana Farber Cancer Institute, Boston, Massachusetts, United States of America; 6 Department of Pediatrics, Cincinnati Children’s Hospital Medical Center, Cincinnati, Ohio, United States of America; University of Alabama at Birmingham, UNITED STATES

## Abstract

Tuberous sclerosis complex (TSC) is an autosomal dominant tumor suppressor gene syndrome due to germline mutations in either *TSC1* or *TSC2*. 10–15% of TSC individuals have no mutation identified (NMI) after thorough conventional molecular diagnostic assessment. 53 TSC subjects who were NMI were studied using next generation sequencing to search for mutations in these genes. Blood/saliva DNA including parental samples were available from all subjects, and skin tumor biopsy DNA was available from six subjects. We identified mutations in 45 of 53 subjects (85%). Mosaicism was observed in the majority (26 of 45, 58%), and intronic mutations were also unusually common, seen in 18 of 45 subjects (40%). Seventeen (38%) mutations were seen at an allele frequency < 5%, five at an allele frequency < 1%, and two were identified in skin tumor biopsies only, and were not seen at appreciable frequency in blood or saliva DNA. These findings illuminate the extent of mosaicism in TSC, indicate the importance of full gene coverage and next generation sequencing for mutation detection, show that analysis of TSC-related tumors can increase the mutation detection rate, indicate that it is not likely that a third TSC gene exists, and enable provision of genetic counseling to the substantial population of TSC individuals who are currently NMI.

## Author Summary

Tuberous sclerosis complex (TSC) is a human genetic disorder due to mutations in the *TSC1* or *TSC2* genes. A mystery for many years has been the fact that with standard genetic testing 10–15% of TSC patients have had no mutation identified (NMI) in either *TSC1* or *TSC2*. We examined the genetic cause of TSC in patients who were ‘NMI’ after previous testing. We found a mutation in *TSC1* or *TSC2* in the vast majority of the subjects studied: 45 of 53 (85%). The majority of mutations identified were either in introns or mosaic or both. Usually we expect to find mutations causing human disease in exons, coding parts of genes. However, mutations can also be found in introns, the non-coding parts of genes, and we found intronic mutations in 18 of 45 subjects (40%). Mosaic mutations were seen in 26 of 45 subjects (58%). Mosaicism is the situation in which different cells in the body have a different genetic make-up, and in this case the mutations in *TSC1/TSC2* were present in only a fraction of the cells from the patient. So these two types of hard-to-find mutations (in introns and/or mosaic) explain the majority of TSC patients who were NMI.

## Introduction

Tuberous sclerosis complex (TSC (MIM: 191100, 613254) [1]) is an autosomal dominant disorder affecting one in 6,000 live births and is characterized by the growth of benign tumors in multiple organ systems and a highly variable phenotype [2,3]. Affected organs include the skin, brain, eyes, heart, kidneys and lungs. In patients who meet standard clinical criteria for TSC [4], pathogenic mutations in *TSC1* or *TSC2* are found in 75–90% of cases [5–17]. *TSC1* is located on chromosome 9q34 and consists of 53,273 nt; *TSC2* is on chromosome 16p13 and and consists of 41,255 nt. The 23 exons of *TSC1* are transcribed into a 8.6 kb transcript (NM_000368.4), and *TSC2* consists of 42 exons encoding a 5.5 kb transcript (NM_000548). Approximately 60 to 70% of TSC cases are sporadic, which reflects a high spontaneous mutation rate in these genes [18–20]. However, 10 to 15% of TSC patients have no mutation identified (NMI), despite thorough molecular diagnostic testing, including exon-based Sanger sequencing and analysis for large genomic deletions in *TSC1* and *TSC2*. NMI TSC subjects generally have milder clinical features of TSC than patients with identified *TSC2* mutations [10,15,21,22]. A molecular understanding of disease pathogenesis in TSC NMI patients has clear importance both for our understanding of genetic mechanisms causing TSC, and for provision of genetic counseling to TSC NMI patients.

We hypothesized that there were several possible reasons for NMI status in TSC patients: 1) mutation detection failure due to technical issues; 2) mosaicism for mutations in *TSC1* or *TSC2*; 3) mutations in introns that affect splicing and are not near exons, or mutations in promoter and enhancer regions of *TSC1* and *TSC2*, regions typically not examined during molecular diagnostic assessment; 4) occurrence of a third TSC gene.

Here we report the results of next generation sequencing analysis of the entire genomic extent of *TSC1* and *TSC2* in 53 TSC NMI subjects using blood and saliva DNA samples as well as biopsies of TSC cutaneous tumors. We identified pathogenic mutations in 45 (85%) patients. Mosaicism was observed for the majority (26 of 45, 58%) of these mutations, ranging from 0.21 to 34% mutant allele frequency. Splice site variants were identified in 18 (40%) subjects. TSC skin tumor biopsy samples yielded a higher rate of mutation detection than blood or saliva samples.

## Results

### Strategy for mutation detection and confirmation

We employed a series of techniques using next generation sequencing (NGS) to identify mutations in *TSC1* and *TSC2*. DNA samples from 53 subjects with TSC and no mutation identified (NMI) by prior studies were studied, including DNA derived from blood and/or saliva from 52 patients, one sample from a fetus who died of TSC complications, and 10 biopsies of TSC skin lesions. 46 subjects had no family history of TSC (sporadic cases), and seven had other family members affected by TSC. NGS was performed on 40 DNA samples using libraries derived from long range PCR amplification of most of *TSC1* and *TSC2* [23]. NGS was also performed on 26 DNA samples (13 of these had also been analyzed by the first method) using libraries derived from hybrid capture of all of *TSC1* and *TSC2* with 10 kb of upstream and downstream sequences, as well as coding regions of *DEPTOR*, *PRAS40*, *TBC1D7*, *DEPDC5*, *NPRL2*, and *NPRL3*. Probable mutations were identified (see Methods for details) and were validated as being real (not sequencing artifacts) through secondary analyses in all cases. Intronic variants were validated as having effects on splicing in studies described below. Heterozygous mutations in sporadic subjects were validated as occurring *de novo* by comparison with parental DNA samples. After these studies confirming both the occurrence of a sequence variant and its likely or confirmed functional effects, mutations were considered definite.

### Summary of findings

**Fig 1:**
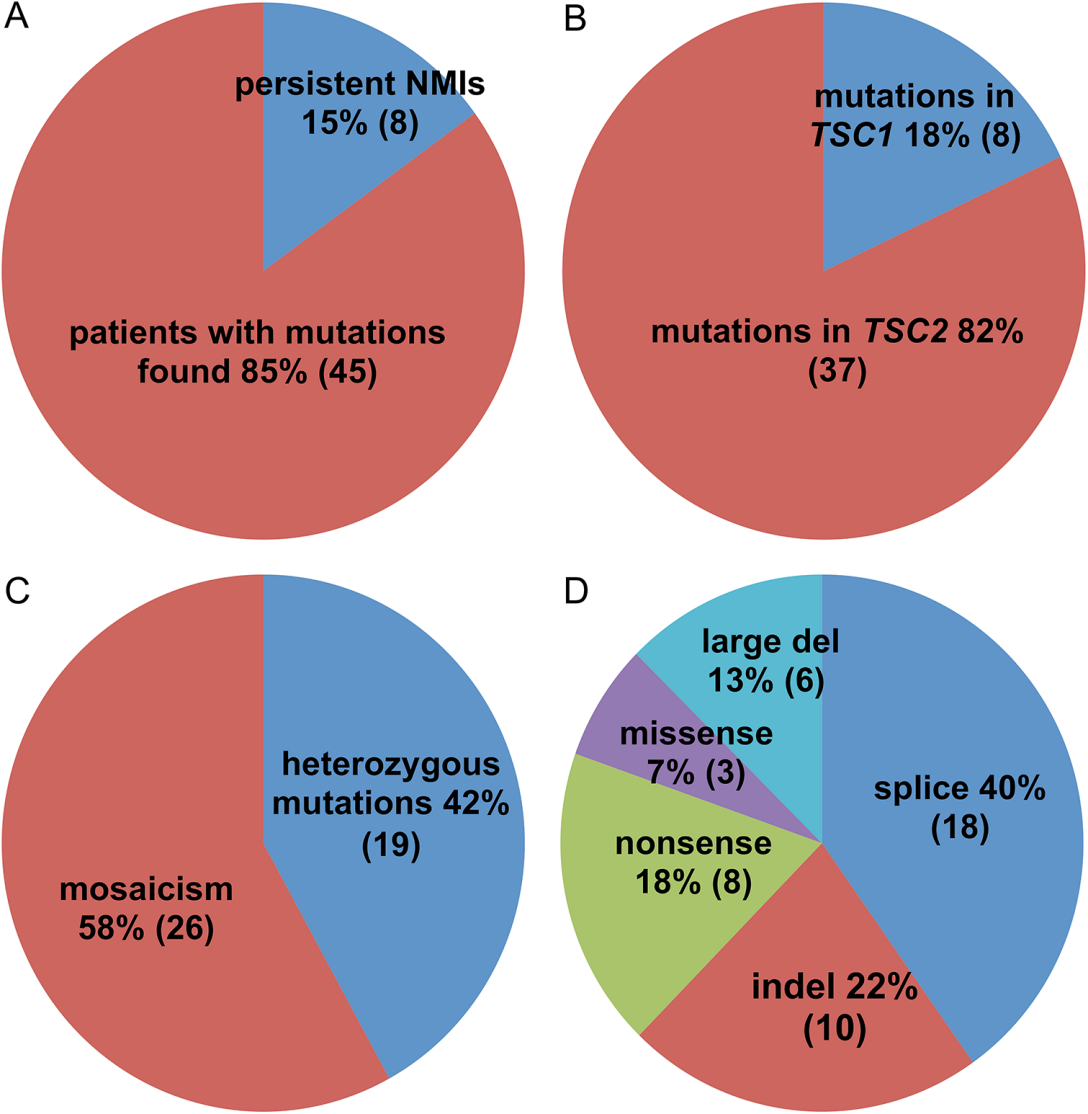
Pie charts displaying the mutation types and frequencies in 53 TSC NMI subjects. (A) Proportion of subjects with mutations identified vs. remaining as persistent NMI. (B) Proportion of mutations in *TSC1* vs. *TSC2*. (C) Proportion of heterozygous vs. mosaic mutations. (D) Different types of identified mutations.

Mutations were identified in 45 of 53 NMI subjects (85%) (Table 1 and Fig 1). Mutations in *TSC2* were found in 37 (82%) patients and in *TSC1* in 8 (18%) patients, a distribution which is similar to that seen in general in TSC [24]. Heterozygous non-mosaic mutations, seen at allele frequency of 40–60% by NGS, were identified in coding exons and consensus splice sites in six of 53 (11%) NMI patients (Table 1: P3, P11, P21, P25, P43, and P52), apparently missed by previous analyses. We did not identify any pathogenic variants in *DEPTOR*, *PRAS40*, *TBC1D7*, *DEPDC5*, *NPRL2*, and *NPRL3* in the analyzed samples. These genes were analyzed as being potential candidates for TSC3, due to their roles in the mTOR signaling pathway.

**Table 1:** Mutations identified in the TSC NMI subjects.

Subject	Gene	Mutation	Mutation type	Mutant allele frequency	tissue types with mutation identified	Age	Sex	Organs affected and # of major features			
								S	B	H	K/L
Variants known as deleterious with 50% AF											
P3f	*TSC2*	c.848+1G>A ^A^	splice	50%	Blood, saliva	31y	F	4	2		1
P10	*TSC2*	c.226-12T>A ^C^	splice	50%	Blood, saliva	5y	F	2	1		
P11	*TSC2*	c.4660C>T; p.Q1554*	nonsense	50%	Blood	8y	F	4	3	1	1
P18	*TSC2*	c.976-15G>A ^B^	splice	50%	Blood, saliva	2y	F	1	1		
P21	*TSC2*	c.5238_5255delCATCAAGCGGCTCCGCCA	indel	50%	Blood, saliva	24y	M	4	4	1	1
P25f	*TSC1*	c.1498C>T; p.R500*	nonsense	50%	Saliva	19y	F	2	2		
P29	*TSC1*	c.-234-?_*1+?del del ex.1_ex.23#	large del	50%	Blood	26y	F	3	3	1	
P33	*TSC2*	c.-106-722_-85del; 744nt del including part of exon 1	large del	50%	Saliva	8y	M	2	2	1	1
P37	*TSC2*	c.976-15G>A ^B^	splice	50%	Blood	7y	F	1	2	1	1
P38f	*TSC1*	c.664-15A>G ^B^	splice	50%	Saliva	1y	M	1	2		
P43	*TSC2*	c.1432_1436delCAGTT	indel	50%	Saliva	27y	F	3	2		1
P46	*TSC2*	c.481+5G>A ^B^	splice	50%	Blood, saliva	13y	F	3	2	1	1
P52	*TSC2*	c.5018_5020delCA	indel	50%	Blood, saliva	1y	M	1	2	1	
Novel or previously recognized variants with uncertain pathogenic effect, with 50% AF											
P1	*TSC2*	c.849-15G>A ^E^	splice	50%	Blood	5y	M	2	3	1	1
P24f	*TSC1*	c.913+3A>T ^E^	splice	50%	Blood, saliva	10y	M	2	1		
P34	*TSC2*	c.3611-9_3611-34delinsCC ^D^	splice	50%	Blood, saliva	22y	F	3	3		1
P35	*TSC2*	c.1717-23T>G ^D^	splice	50%	Blood	14y	M	2	1	1	
P36f	*TSC2*	c.1362-10C>A ^E^	splice	50%	Saliva	30y	M	2	2		
P41	*TSC2*	c.2221-126C>T ^D^	splice	50%	Blood, saliva	15y	F	2	4		
Mosaic variants with AF <50%											
P8	*TSC2*	c.976-15G>A ^B^	splice	34%	Saliva	7y	F	3	2		1
P53	*TSC1*	c.1776delG	indel	30%	Blood, saliva	22y	M	3	2		1
P12	*TSC2*	c.3610+1G>T ^A^	splice	22%	Blood	4y	M	1	2		
P39	*TSC2*	c.3884-?_PKD1c.11270-?del^b^	large del	17%	Blood, saliva	18y	F	4	2		
P2	*TSC1*	c.2689C>T; p.Q897*	nonsense	15%	Blood	20y	M	3	3	1	
P4	*TSC2*	c.4943T>C; p.I1648T	missense	8.1%	Blood	9y	M	2	2	1	
P49	*TSC2*	c.848+281C>T ^B^	splice	6.9%	Angiofibroma, blood, saliva, normal skin	33y	M	1	1		
P22	*TSC2*	c.226-12T>A ^C^	splice	5.9%	Fetal tissue	fetus			1	1	
P30	*TSC2*	c.1413_1414delTG	indel	5.3%	Blood, normal skin	33y	M	2			1
P6	*TSC1*	c.2111_2112delAT	indel	4.7%	Blood, normal skin	4y	M	1	2	1	
P45	*TSC2*	c.4051G>T; p.E1351*	nonsense	4.4%	Saliva	19y	F	2			1
P26	*TSC2*	c.1717-247_ *CASKIN1*c.1417-380del; 114,113 bp del intr.15_ *CASKIN1*intr.14	large del	4.0%	Saliva	6y	F	2	3		
P5f	*TSC1*	c.2374C>T; p.Q792*	nonsense	3.3%	Blood	57y	F	2			1
P15	*TSC2*	c.4238insGGTTAAG	indel	3.2%	Blood	40y	F	2	1		2
P9	*TSC2*	c.1717-4103_c.3356del; 13,282 nt del intr.15_ex.28	large del	3.0%	Saliva	1y	F	1	2	1	
P27	*TSC2*	c.2356_2362delCGCGAGA	indel	3.0%	Blood, ungual fibroma, angiofibroma, normal skin	34y	M	2	3		1
P28	*TSC2*	c.2098-86_4493+31del; 12,591nt del intr.18_intr.33	large del	3.0%	Saliva	8y	F	3	3		1
P16	*TSC2*	c.1108C>T; p.Q370*	nonsense	2.7%	Blood, saliva	2y	M	1	2	1	
P13	*TSC2*	c.3685C>T; p.Q1229*	nonsense	1.7%	Blood	14y	M	1	3		
P20	*TSC2*	c.5228G>A; p.R1743Q	missense	1.4%	Blood	30y	M	2	1		1
P31	*TSC2*	c.1831C>T; pR611W	missense	1.2%	Saliva	18y	F	2	1	1	1
P32	*TSC2*	c.4850-1G>A ^A^	splice	0.71%	Blood	17y	F	3	2	1	
P17	*TSC2*	c.2647C>T; p.Q883*	nonsense	0.69%	Blood, angiofibroma	2y	F	1	1	1	
P51f	*TSC2*	c.4180_81delCT	indel	0.21%	Blood, saliva, angiofibroma (2), daughter’s blood	34y	F	1	1		1
P14	*TSC2*	c.976-15G>A^^^	Splice	-	Angiofibroma, normal skin	23y	M	1	1		1
P42	*TSC2*	c.4530_4537delTGGCGACG^^^	Indel	-	Angiofibroma (3), normal skin	25y	F	1	1		1
Subjects with no mutation found											
P7		no mutation found				11y	M	1	1		
P19		no mutation found				33y	F	2			1
P23		no mutation found				9y	M	1	1		
P40		no mutation found				33y	M	4	2		1
P44		no mutation found				20y	F		2		1
P47		no mutation found				1y	M	1	3		
P48		no mutation found				4y	M	1	2		
P50		no mutation found				23y	F		1		1

Column 1 is subject number. f means that this was a familial case of TSC. All others had no family history of TSC.

Column 3, letters next to splice site variants

^A^ variants affecting canonical splice sites

^B^ reported as affecting splicing and / or being pathogenic

^C^ aberrant TSC2 transcripts shown in this study

^D^ high molecular weight product by RT-PCR of RNA, or allelic distortion of exonic SNPs by RT-PCR analysis

^E^ intronic variants not seen previously, that were de novo (n = 1) or segregated with TSC (n = 2), without other confirmatory evidence of effect on splicing.

^^^In P14 and P43 mutations were detected in skin lesions but not in blood or saliva samples (see S2 Table).

^#^ Identified by MLPA.

Column 7 gives the age of the subject at the time of collection of clinical data.

Columns 9–12 indicate the presence or absence of involvement of Skin (S), Brain (B), Heart (H), Kidneys (K), Lungs (L). A blank entry in these columns means no involvement of the respective organ; a number entry indicates the number of major TSC manifestations present in the subject for that organ.

### Mosaicism and analysis of skin biopsies

Mutations were mosaic in 26 of 45 (58%) subjects in whom mutations were identified, ranging from 0.21% to 34% mutant allele frequency (AF) (Table 1 and Fig 1 and S2 Table). Seventeen subjects had mosaicism with very low mutant AF, < 5%. These mutations consisted of five indel, five nonsense, two splice site, two missense, and three genomic deletion mutations. All were confirmed by secondary analyses (S2 Table) using SNaPshot single nucleotide sequencing, amplicon NGS, or PCR with sequencing across the deletion fusion junction.

Here we briefly review each case in which a mutation was identified at AF < 1%. Subject P17 was found to have a nonsense *TSC2* p.Q883* mutation in initial blood DNA analysis by NGS with long-range PCR in 10 of 1,806 reads (0.55%, S2 Table). Subsequent amplicon NGS of blood and angiofibroma DNA showed an AF of 0.69% and 0.77%, respectively, while this sequence variant was seen at an average rate of 0.11% (range 0.07–0.16%) in 25 control samples.

We identified a *TSC2* c.4850-1G>A splice site variant in a blood sample from subject P32 by initial NGS analysis in 41 of 5,745 reads (0.71%, S2 Table). The mutation was confirmed in this sample by targeted amplicon NGS in 2663 of 955,336 reads (0.28%, S2 Table), with an average mutant AF of 0.09% (range 0.02–0.20%) seen in 25 control samples.

Subject P51 was a mother whose daughter also had been diagnosed with TSC, with a *TSC2* c.4180_81delCT heterozygous mutation identified in her blood sample by conventional testing and not present in the mother. Amplicon NGS of several samples from the mother demonstrated that the c.4180_81delCT mutation was seen at 0.98% and 1.4% AF in two different angiofibromas, at 0.07% and 0.21% in blood and saliva, respectively, and at an average 0.005% (range 0–0.03%) frequency in 25 controls (S2 Table). These data indicate that the mother has extreme low level mosaicism, both somatic and germline, for this *TSC2* indel mutation, which she transmitted to her daughter.

In two subjects, skin biopsy analyses demonstrated mutations in *TSC2*, which could not be seen in the analysis of blood or saliva DNA (S2 Table). For P42, an angiofibroma was found to have both an indel (c.4530_4537delTGGCGACG) and a missense (p.L590R) mutation in *TSC2* at 4.6% and 2.7% AF, respectively. Amplicon NGS of the original and two additional angiofibroma showed that all three had the indel allele at 0.86% to 2.3% AF, while the missense allele was seen at significant levels only in the original angiofibroma. Both variants were seen at extremely low frequency (< 0.02%) in blood and saliva DNA from this subject, suggesting that this individual had extreme germline mosaicism for the indel mutation. For P14, analysis of an angiofibroma led to identification of three mutations in *TSC2* at AF from 1.3% to 8.9% (S2 Table). Amplicon NGS confirmed these findings, but also demonstrated that the allele frequency of the three mutations in saliva and blood was similar to normal controls. Two of the three mutations were observed at higher AF in normal skin (0.61% and 0.63%), suggesting that one or the other was an extreme mosaic germline allele present in this individual. We suspect that the splice site variant (c.976-15G>A) seen at 8.9% frequency in the original angiofibroma is the mosaic germline allele, and the other two variants (p.P1497L and p.R458*) are second hit mutations that led to development of two separate angiofibroma clones that collided on this patient’s face.

Two other subjects (P27 and P49) also provided both skin and saliva/blood DNA for analysis (S2 Table). In both cases the mutant AF was higher in the skin biopsies than in saliva or blood DNA, similar to the two subjects above, and suggestive of enrichment in the skin biopsy for cells bearing the mosaic germline mutation. This was not observed in subject P17 (described above), consistent with the possibility that the biopsy was not a true TSC tumor or that the biopsy was too superficial and did not include the dermis where aberrant *TSC2*-mutant fibroblasts are known to reside [23].

### Splice site mutations

**Fig 2:**
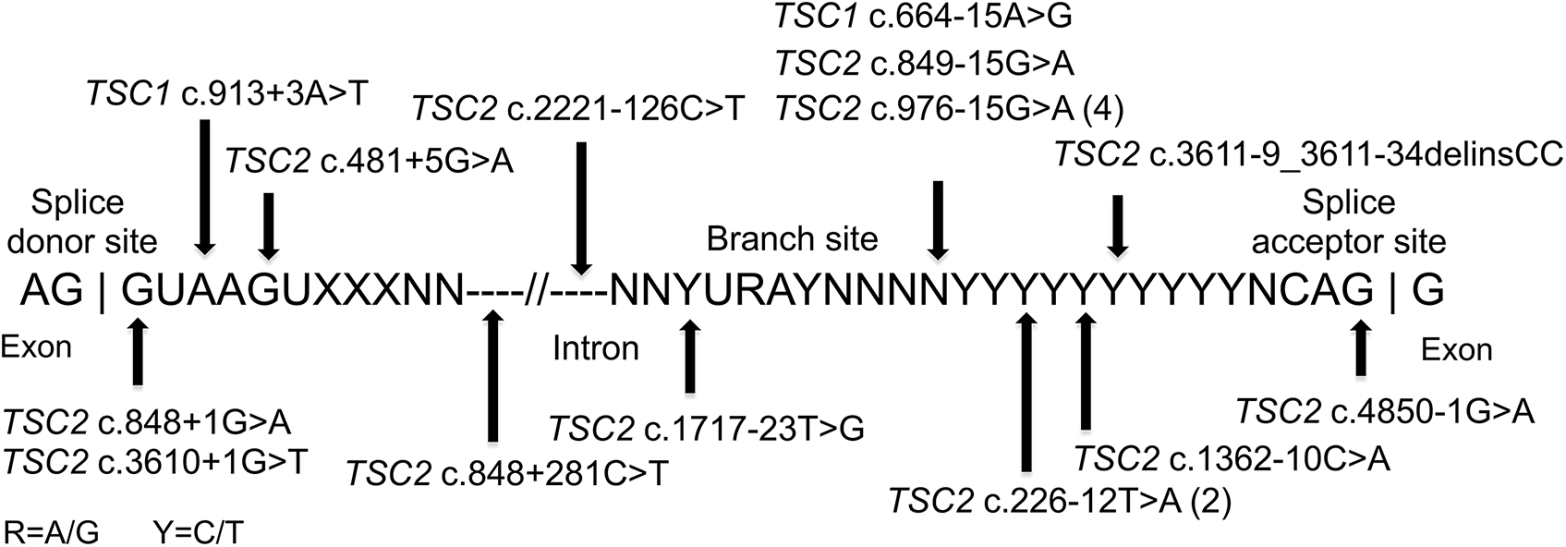
Intronic mutations in 18 TSC NMI subjects. The locations of 18 splice site mutations identified are shown relative to the canonical consensus sequences present at the 3’ exon region, the branch site, and the 5’ exon region.

Mutations affecting splicing were unusually common in this set of NMI subjects, compared to previous reports of TSC mutation analysis (9.5% and 16.2% of all mutations in *TSC1* and *TSC2*, respectively) [3,25], seen in 18 of 45 (40%) subjects with mutations identified. Intronic mutations were identified in canonical sites near exons (three), near branch point sites (seven), at other locations near exons (six), and at deep intronic locations (two) (Fig 2). As above, family studies demonstrated that all of these mutations were strictly associated with disease status, with 15 occurring in sporadic TSC cases, not seen in parental samples. Maternity and paternity was confirmed for parents of subjects P1, P35, and P41, in whom novel splice site variants were identified. In addition to the three subjects with mutations in canonical splice sites (P3, P12, P32), seven subjects each had one of four splice mutations that had previously been reported to be pathogenic: two mutations seen in five subjects had been reported previously as affecting splicing [26] (*TSC2* c.976-15G>A (P8, P14, P18, and P37), and *TSC2* c.848+281C>T (P49)); and two subjects with distinct splice mutations reported as pathogenic [27] (*TSC1* c.664-15A>G in P38, and *TSC2* c.481+5G>A in P46). Lymphoblastoid cell lines (LCLs) were prepared for the remaining eight (seven unique) putative intronic variants to enable functional confirmation. RT-PCR analysis of one sample, P10, displayed aberrant bands on agarose gel, confirmed by sequencing as corresponding to skipping of exons 3–5 (S1A Fig). The same mutation (*TSC2* c.226-12T>A) was also present in P22. A high molecular weight smear was seen in RT-PCR analysis of P34 (*TSC2* c.3611-9_3611-34delinsCC, S1B Fig), suggesting aberrant splicing. Other samples with intronic variants did not show aberrant bands in RT-PCR analysis. However, RT-PCR analysis of non-puromycin treated LCLs from 2 cases (P35 *TSC2* c.1717-23T>G, and P41 *TSC2* c.2221-126C>T) with exonic SNPs showed that there was allelic distortion (SNP allele ratio 0.18 and 0.43 for the alleles at lower frequency) consistent with effects on splicing leading to nonsense-mediated mRNA decay (S2 Fig). Allelic distortion with relative loss of the mutant allele (allelic ratio 0.26–0.79 (range), median 0.54) due to nonsense-mediated mRNA decay has previously been reported for indel, nonsense, and splice mutations in TSC1 using TSC lymphoblastoid cell line RNA preparations [28]. In addition, analysis of the GTEx data set of RNA-Seq data for 2,387 samples from 155 individuals heterozygous for the *TSC2* coding region synonymous SNP rs1748 and 52 different tissue sites demonstrates that the allele ratio is centered at 0.89, with 99% of values > 0.43 (K. Ardlie and T. Sullivan, GTEx project, dbGAP phs000424.v6.p1). Three subjects (P1, P24, and P36) had no exonic SNPs in genes with intronic variants identified, so this analysis could not be performed.

### Large genomic deletions

Large genomic deletions were identified in *TSC2* in three subjects by analysis of insert size in the hybrid capture NGS data (S3 Fig). These were seen in subjects: P9–13,282 nt deletion at 3% AF; P26–114,113 nt deletion at 4% AF; and P28–12,591 nt deletion at 3% AF. All of these were verified by PCR across the deletion junction and sequencing.

### Genotype-phenotype correlations

**Fig 3:**
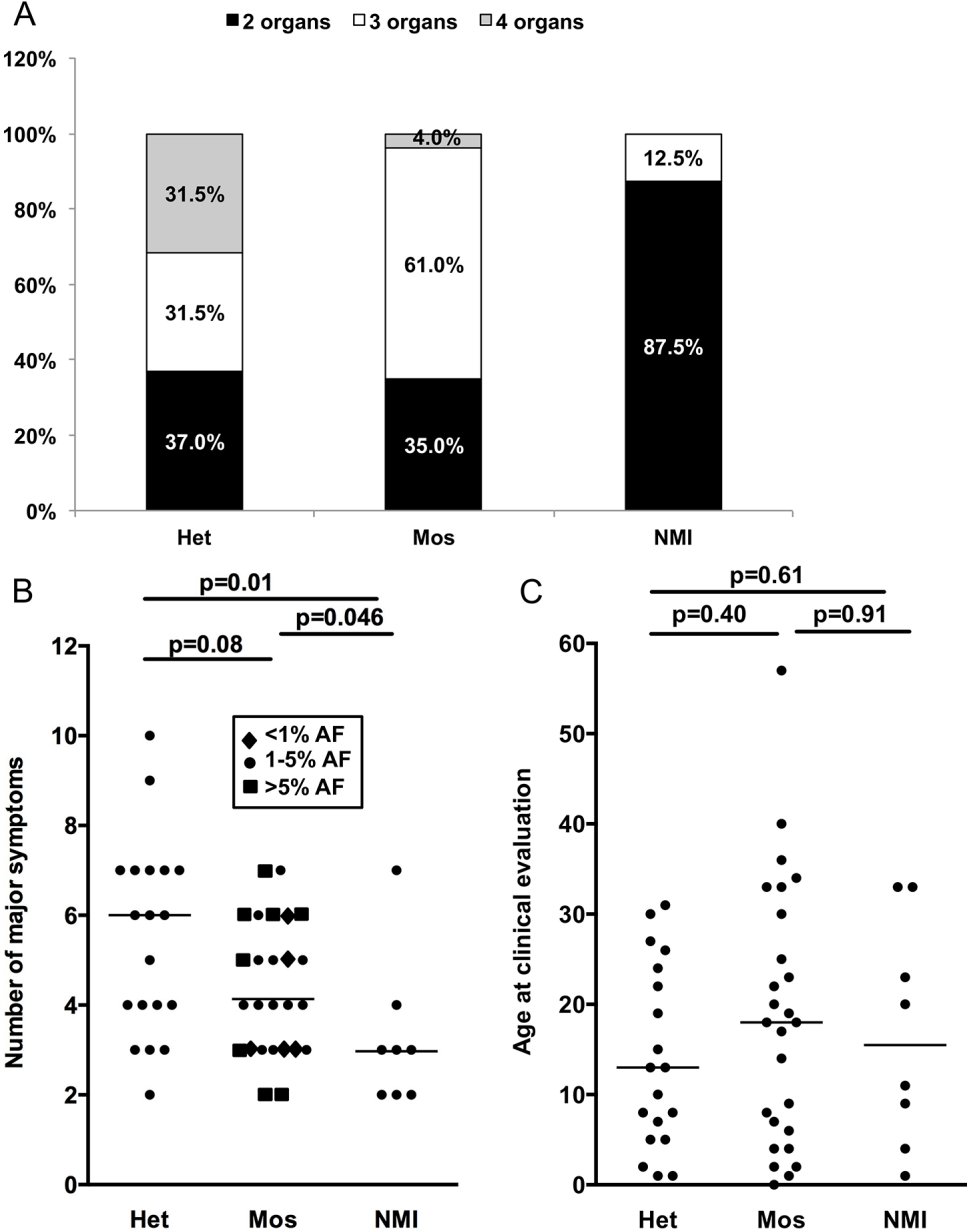
Correlation between clinical features and mutation status in 53 NMI subjects. (A) The proportion of subjects with 2, 3, or 4 organs affected; with heterozygous or mosaic mutations, or persistent NMI status. P = 0.003. (B) The number of major symptoms seen for each subject sorted according to mutation status. Note that differing levels of mosaicism have different symbols according to allele frequency (AF). (C) Age at the time of clinical evaluation, sorted according to mutation status. Het, heterozygous; Mos, mosaic. P values determined by chi square test (A) and Mann-Whitney unpaired test (B and C). Results with p < 0.05 are considered statistically significant.

Clinical information on TSC symptoms was used to compare clinical phenotype among subjects with different mutation detection status: heterozygous, mosaic, and persistent NMI. (Note that we use the term persistent NMI to refer to subjects in this study who continued to have no mutation identified after all of our analyses were completed.) We considered both the number of organ systems involved (among skin, brain, cardiac, and renal/lung), and the number of TSC manifestations that are major criteria for diagnosis [4]. Six of seven subjects with four organ systems involved had heterozygous mutations, while one had mosaicism (1.2% AF), and none were persistent NMI (p = 0.003, Fig 3A). Furthermore seven of eight (87.5%) persistent NMI subjects had only two organ systems involved in contrast to both heterozygous and mosaic mutation categories, for which two organ system involvement was seen in the minority: 37% and 35%, respectively (Fig 3A). Similarly, the number of major TSC manifestations was a median of six for the heterozygous mutation subjects, four for the mosaic mutation subjects, and three for those with persistent NMI, with statistically significant differences in these numbers between persistent NMI and the heterozygous mutation subjects (p = 0.01), and between persistent NMI and the subjects with mosaicism (p = 0.046; Fig 3B). Since TSC manifestations often appear as patients with TSC age, we also examined the correlation between mutation status and age. There was not a significant difference in age ranges among these three categories of mutation status (Fig 3C), suggesting that a difference in age did not explain the correlation between disease severity and mutation detection status.

## Discussion

Here we report on analysis of 53 TSC patients who had no mutation identified after conventional molecular diagnostic analysis of *TSC1* and *TSC2*. We studied both blood and saliva DNA samples, and had one or more skin biopsies of TSC tumors from six subjects. We identified mutations in 45 of 53 subjects (85%). Mosaicism was seen for these mutations in the majority (26 of 45, 58%). Intronic mutations were unusually common, identified in 18 of 45 subjects (40%). Six subjects had coding region or consensus splice site mutations at full heterozygous allele frequency, apparently missed in earlier analyses.

Five of six subjects in whom skin tumors were biopsied for genetic analysis demonstrated a higher prevalence of the mutation in the skin biopsy than in blood or saliva DNA. This was expected given the hypothesis that the clonal fibroblasts giving rise to the skin tumor would have originated from the cells bearing the initial mosaic mutation. Secondary mutations were observed in all three of the skin tumors in which full NGS was performed, consistent with point mutation as a second hit event to eliminate normal function of *TSC2*. In one subject (P17), there was no enrichment for the mutation in the skin tumor biopsy, possibly due to the biopsy not penetrating the dermis (the site of the mutant fibroblasts), or the lesion not being a true TSC-related tumor.

Deep intronic mutations have been identified in multiple genes causing Mendelian disorders, and in some cases make a significant contribution to the total mutation burden [29–31]. Further, several deep intronic mutations have been identified in TSC, all involving *TSC2* [26,32,33]. In this study, we identified intronic mutations in 18 subjects, of which 15 did not affect nucleotides in canonical splice site motifs. Intronic mutations seen in five of these 15 subjects (33%) were completely novel, to our knowledge, while the mutations in the remaining 10 had been seen previously, although several did not have definitive information in regards to pathogenicity. Splice site mutations overall account for about 9% of all *TSC1* mutations and 16% of all *TSC2* mutations [3], whereas they accounted for 40% of the mutations identified in this set of NMI subjects, indicating that they are a main contributor to lack of mutation identification in the TSC NMI population.

Somatic (generalized) and germline (confined gonadal) mosaicism for *TSC1* and *TSC2* mutations have been described in many TSC patients and their parents, respectively [15,16,20,34–41]. Mosaicism was reported in 26% and 15% of TSC patients from two series who had large genomic deletions and rearrangements in *TSC1* or *TSC2* [41,36]. However, somatic mosaicism has appeared to be much less common than this for small mutations in *TSC1* and *TSC2* [10,15,34,38,42–44]. Indeed our previous analysis of TSC NMI subjects using an early NGS technique identified mosaic mutations in only two of 38 (5.3%) [45]. Here we identified mosaic mutations in 26 of the NMI subjects studied, of which 17 (65%) were observed at an allele frequency < 5%, five at an allele frequency < 1%, and two were identified in skin tumor biopsies only and were not seen at appreciable frequency in blood or saliva DNA. These results indicate that mosaicism is much more common in TSC than previously appreciated. The majority of mosaic mutations would have been missed using conventional Sanger sequencing or NGS with low read depth. The occurrence of what we call ‘extreme mosaicism’ with representation of the mutant allele at less than 1% in saliva or blood DNA is particularly notable. Mosaicism is well-known in many tumor suppressor gene syndromes, and has been described in particular detail for NF2 [46]. More than 25% of sporadic cases of NF2 are mosaic, with the mutation often detected only in tumor material and not in lymphocyte DNA [47,48]. Since the NMI category has represented about 15% of the TSC population in the past, our finding of mosaic mutations in half (26 of 53) of these individuals suggests that mosaicism occurs in at least 7.5% of individuals presenting with TSC.

The association we noted between mutation (heterozygous vs. mosaic vs. persistent NMI status) and phenotype obviously fits with expectations, in that lower prevalence of a TSC gene mutation in an individual would be expected to reduce both haplo-insufficiency effects and propensity for second hit bi-allelic inactivation, leading to fewer tumors and organ systems involved. Indeed, the occurrence of diagnosable TSC in individuals with extreme mosaicism is perhaps surprising. However, the prevalence of the mutant allele in critical tissues susceptible to second hit tumor induction during development cannot be estimated from the tissue samples available to us.

We recognize that there are several potential limitations to our study, and that some of our data should be interpreted with caution. First, the number of subjects that we have studied is not large, although substantial. The phenotype comparisons we have made among subjects according to mutation status (heterozygous vs. mosaic vs. persistent NMI status) are based upon analysis of relatively small subsets, and though statistically significant, should be considered in that light. Second, an ideal standard for confirmation of mosaicism in a patient might include: confirmation of a mosaic mutation by two different methodologies; and/or confirmation of mosaicism in two or more tissue samples. In this study, 14 of 26 (54%) mosaic variants were validated by an alternative technology on a single sample: 5 small mutations validated by Sanger; 6 small mutations validated by SNaPshot; and 3 genomic deletions validated by PCR across the deletion junction followed by Sanger sequencing. 7 of 26 (27%) mosaic variants we identified were seen in more than one sample, and 4 of the 7 were seen in 3 or 4 samples (S2 Table). For the remaining 5 of 26 (19%) mosaic variants, only a single sample was available, and findings were validated by amplicon NGS (S2 Table). So, 21 of 26 (81%) of the mosaic variants we detected were confirmed according to these ideal standards. Third, mutation findings seen at < 1% AF or in skin lesion biopsies only should be considered cautiously, though we did perform confirmatory analyses in each of these cases. As indicated in S2 Table, variant nucleotides were seen at low frequency for control samples (maximum 0.43%, more commonly in the range of 0 to 0.3%) due to the intrinsic noise that occurs in next generation sequencing. However, the mutant AF was always higher in the subject samples (S2 Table). In addition, the finding of the same mutation in the TSC parent (P51) at low allele frequency (0.07% and 0.21% in blood and saliva) who had an affected child at full heterozygote frequency, provides strong evidence that mutations seen at AF < 1% are real at least in some cases. Fourth, we may be overstating our success rate in mutation identification by retention of subjects with various mutations that should or might have been found by previous analyses, including the single individual (P29) with a *TSC1* genomic deletion, all those with heterozygote mutations in exons or canonical splice sites (n = 6), non-canonical intronic mutations previously known to be pathogenic (n = 4), and mosaic mutations seen at AF > 15% (n = 5). However, we opened this study to any TSC individual who was in the category of NMI by prior study, and scrutinized laboratory testing reports to ensure that complete testing had been performed. Hence we feel that it is most appropriate to retain all subjects in consideration of success rate of mutation identification and overall findings. If these subjects are excluded from consideration, then our success rate of mutation identification becomes 29 of 37 (78%), which is still substantial. Fifth, the three intronic mutations we identified for which we could not identify aberrant RT-PCR products by gel electrophoresis or sequencing should be considered somewhat tentative, even though we showed that they had occurred de novo, with confirmation of paternity and maternity for parental DNA samples.

In summary, we were able to identify a *TSC1* or *TSC2* mutation in the vast majority of TSC individuals who had no mutation identified in previous studies. There are several implications. First, it is very unlikely in our view that there is an additional gene that causes typical TSC. It seems much more likely that those individuals who were persistent NMI are in the extreme mosaic category and NGS of TSC-related tumors from these subjects could result in mutation detection. Second, comprehensive analysis of the *TSC1* and *TSC2* loci is required to have robust mutation identification in TSC, and NGS analysis with high read depth (> 500x) across the coding region and flanking intronic regions (minimum 50 nt) is required to have high sensitivity for detection of mosaicism. Analysis of each entire gene (including intron and flanking regions) by hybrid capture is also required for detection of low mosaic genomic deletions through analysis of paired end reads, of which three were seen in this series. Third, analysis of TSC-related tumors is an effective approach to increase the ability to detect mutations in *TSC1/TSC2* in TSC individuals. Fourth, use of these strategies will lead to more effective genetic counseling for a substantial fraction of TSC individuals in whom past studies failed to identify a causative mutation.

## Materials and Methods

### Ethics statement

All patients and/or their parents provided written informed consent for this research, and the study was approved by the Partners Human Research Committee, the Institutional Review Board for the Partners Hospitals (1999P010781 and 2013P002667).

### TSC subjects

Patients with TSC and NMI status were enrolled between 2010 and 2015 at Boston Children’s Hospital, Cincinnati Children’s Hospital Medical Center, Massachusetts General Hospital, and by an open call for patients through the Tuberous Sclerosis Alliance. All patients and/or their parents provided written informed consent for this research, and the study was approved by the Partners Human Research Committee, the Institutional Review Board for the Partners Hospitals.

52 subjects provided blood and/or saliva samples for DNA extraction, and DNA from a fetus that died due to TSC complications was provided in one case. All patients met standard clinical criteria for the diagnosis of TSC [4], and had been previously studied by Sanger sequencing of *TSC1* and *TSC2* exons and deletion/amplification analysis of *TSC2*, with no mutation identified. Reports of molecular testing for all subjects were obtained and directly reviewed to confirm that full testing had been performed.

Demographic and clinical data were collected for all subjects. Both medical record review and self-reporting from subjects was used to assess the presence or absence of the following TSC features (grouped by organ system): skin (three or more hypomelanotic macules, three or more facial angiofibromas or forehead plaque, two or more ungual fibromas, shagreen patch); CNS involvement (tubers, subependymal nodules, subependymal giant cell astrocytomas, retinal hamartomas, seizures, infantile spasms, cognitive impairment); cardiac rhabdomyomas; renal angiomyolipomas and cysts, and pulmonary lymphangioleiomyomatosis (LAM) (S1 Table). The four organ systems indicated (skin, CNS, heart, and kidney/lung) were used to assess the number of organ systems affected. Due to evidence that renal angiomyolipoma and LAM have common mutations and a shared pathogenesis [2], they were considered one organ system for this analysis.

Biopsies of skin tumors were obtained from six patients (nine facial angiofibromas and one ungual fibroma) and were also subject to mutational analysis. The skin tumor biopsy study was approved by the Partners Human Research Committee.

### Lymphoblastoid cell transformation and culture

Blood samples from subjects with potential splice site mutations were used to generate Epstein-Barr virus (EBV)-transformed B-lymphoblastoid cell lines at CHGR Tissue Culture Core, Massachusetts General Hospital, Boston. Lymphoblastoid cell lines were cultured in RPMI 1640 with L-Glutamine medium, with 10% FBS and 1% Pen Strep (Gibco) at 37^°^C and 5%CO_2_.

### DNA and RNA analysis

Genomic DNA was isolated from blood and saliva cells using the QIAamp DNA Kit (Qiagen), and from skin samples using the Puregene Genomic DNA Isolation kit (Qiagen). Total RNA was extracted from lymphoblastoid cell line cultures using the RNeasy Mini Kit (Qiagen).

### Examination for large genomic deletions in *TSC1*

Enrolled subjects who had not had previous deletion/amplification analysis of *TSC1* (n = 17) were examined using multiplex ligation-dependent probe amplification (MLPA) assay with probe sets for all exons of *TSC1*, available from MRC-Holland, as described previously [41].

### Next generation sequencing (NGS) and computational analysis

Next generation sequencing of the genomic extent of *TSC1* and *TSC2* was performed as described previously [23,49] on DNA extracted from blood, saliva, and fresh skin samples on the HiSeq2000 or HiSeq 2500 instruments (Illumina). Sequencing libraries were prepared using two methods: long-range PCR followed by fragmentation; and Agilent SureSelect Target Enrichment (Agilent Technologies) capture, followed by use of the KAPA DNA Library Preparation kit. Briefly, long-range PCR (3–8 kb amplicons) was done to amplify all of the coding exons, promoters, UTRs and most of the intronic sequence of *TSC1* and *TSC2* of DNA samples from 40 TSC subjects. Amplicons were purified and used to prepare a small fragment library. Libraries from different samples were generated using unique indices and mixed at an equimolar ratio for sequencing analysis using 50–75 nt paired-end reads at the Partners Center for Personalized Genetic Medicine DNA Sequencing Facility, Cambridge, MA. The Agilent SureSelect hybrid capture bait set covered the entire *TSC1* and *TSC2* genes, including 10 kb upstream and 10 kb downstream, and coding regions of *DEPTOR*, *PRAS40*, *TBC1D7*, *DEPDC5*, *NPRL2*, and *NPRL3*. These libraries were prepared and sequenced at the Center for Cancer Genome Discovery, Dana Farber Cancer Institute, Boston.

For both types of libraries, primary sequence data were deconvoluted using index sequences to individual sample files and converted to FASTQ format, aligned to the human genome using bwa-0.5.8c (Burrows-Wheeler Alignment) [50], filtered to eliminate reads of low quality and to reduce redundancy to a uniform 50 reads starting at each nucleotide position of interest in each direction. The data were then analyzed for sequence variants using tools from the Genome Analysis Toolkit (GATK) [51], including IndelGenotyperV2 and UnifiedGenotyper, to identify indels and single-nucleotide variants. A second approach was used in parallel to analyze the sequence data, with capture of read calls at all positions using SAMtools Pileup [52], followed by custom processing in Python and Matlab to determine base call frequency at each position in each read orientation. These data were then filtered to eliminate variant calls observed in only a single read orientation, or seen in multiple samples to exclude artifacts derived from the sequencing process. All variants observed at a frequency of ≥ 1% were directly reviewed using the Integrative Genomics Viewer [53], to identify bona fide variant calls and exclude sequencing or alignment artifacts. To achieve greater sensitivity for samples in which there were no mutation findings from this initial analysis, the variant frequency cut-off was lowered to 0.5% and three reads for single nucleotide variants (SNVs) and to 0.2% and two reads for indel variants.

The median read depth for coding exons of *TSC1* and *TSC2* was ≥ 5,000x for samples analyzed by long-range PCR, and ≥ 500x for libraries generated by hybrid capture.

Genomic deletions (size > 1kb) were identified in the samples analyzed by hybrid capture using paired end sequencing information to determine the size of the insert fragment. Reads with insert sizes > 1,200 were flagged, and those reads for which insert size was > 2,000 with clustering of at least three reads within a 500 nt region, or for which the insert size was 1,200–2,000 with clustering of at least five reads within a 500 nt region were considered further. All candidate genomic deletions were confirmed by PCR across the deletion fusion junction.

SNVs and indels that were identified as novel and/or of possible significance were confirmed by Sanger bidirectional sequencing when seen at ≥ 15% allele ratio; SNaPshot analysis for those seen at 2–15% allele ratio; and amplicon NGS for those observed at < 2–5% allele ratio. SNaPshot analysis was performed as described previously [45] using ABI Prism SNaPshot Multiplex Kit (Applied Biosystems), analysis of extension products on an ABI 3100 sequencer, and quantification of alleles using GeneMapper version 3.0 (Applied Biosystems).

Targeted short amplicon NGS was performed by NGS analysis of individual amplicons, leading to generation of 10,000–1,000,000 sequencing reads. These read files as well as the primary original read files were interrogated using Unix grep with a 20 nt sequence matching the wild type allele and the mutant allele, to determine the precise frequency of mutant and wild type reads. Similar methods counting the number of reads with normal versus large insert size were used to determine the allele frequency of large genomic deletions.

Missense and potential splice site variants were compared with findings in the LOVD Tuberous Sclerosis mutation database [27] to help assess pathogenicity. Variants observed at any frequency in the 1000 Genomes variant server [54], the NHLBI Exome Variant Server [55], or the ExAC Exome Aggregation Consortium browser [56] were not considered further, as they were likely nonfunctional variants. Only a single novel missense variant was identified, which was predicted to be damaging by PolyPhen2 [57,58] and SIFT [59,60].

MLPA assay with probe sets for all exons of *TSC2*, available from MRC-Holland, was performed in subjects with persistent NMI status, as described previously [41].

### Reverse transcription PCR

RT-PCR was done on RNA prepared from untreated and puromycin-treated (300 μg/ml for six hours) cultures of lymphoblastoid cells using OneStep RT-PCR kit (Qiagen) and primers specific to cDNA regions of *TSC1* and *TSC2*.

### Microsatellite DNA fingerprinting

DNA samples from three sporadic TSC subjects (P1, P35, and P41) and their parents were subject to microsatellite DNA fingerprinting using the following markers: amelogenin, D3S1358, D13S317, D18S51, VWA, D21S11, D7S820, D5S818, D8S1179, and FGA; at the Tissue Typing laboratory, Brigham and Women’s Hospital, Boston to confirm family status. These subjects all had de novo splice site variants at heterozygous frequency.

### Statistical analyses

Statistical comparisons were performed using chi square (*χ*²) test for binary features and Mann-Whitney unpaired test to compare quantitative measures.

## Supporting Information

S1 Fig RT-PCR analyses of intronic mutations. (A) Aberrant bands seen on agarose gel of RT-PCR product of TSC2 exons 1–7 for P10 are shown at left. At right is a Sanger sequencing trace demonstrating that the sequencing becomes duplicated at end of exon 2, and one of the adjacent sequences is that of exon 6. (B) Aberrant high molecular weight smear observed for P34 for RT-PCR amplicons covering exons 28–33 and 29–32 of TSC2 (lanes 1 and 2, respectively). The same amplicons for the control sample do not show this effect (lanes 3 and 4). The splice variant in P34 is located in TSC2 intron 29. RNA was prepared from puromycin-treated LCL cultures for both (A) and (B).
(TIF)

S2 Fig RT-PCR analyses of exonic SNPs demonstrating allelic skewing in RT-PCR products. (A) P35, comparison of genomic DNA (left) and RT-PCR DNA (right, forward and reverse strand). Sanger sequencing demonstrates skewing of the allelic representation of the G and A alleles in the RT-PCR analysis. (B) P41, comparison of genomic DNA (left) and RT-PCR DNA (right, forward and reverse strand). Sanger sequencing demonstrates skewing of the allelic representation of the C and T alleles in the RT-PCR analysis.
(TIF)

S3 Fig IGV screen shots for large deletions detected by NGS. (A) 13,282 nt deletion seen in P9. (B) 12,591 nt deletion seen in P28. Paired end reads from NGS are displayed as boxes connected by a thin line, which represents the inferred insert size.
(TIF)

S1 Table Clinical features of 53 TSC NMI subjects. (PDF)

S2 Table Mutant allele frequency (AF) for mutations confirmed by NGS. (PDF)

S3 Table Primer sequences used in this study. (PDF)
