# Supplementary figures and images for: Mosaic and Intronic Mutations in TSC1/TSC2 Explain the Majority of TSC Patients with No Mutation Identified by Conventional Testing
Source: PLoS Genet. 2015 Nov 5;11(11):e1005637. doi: 10.1371/journal.pgen.1005637 (PMC4634999; doi:10.1371/journal.pgen.1005637)

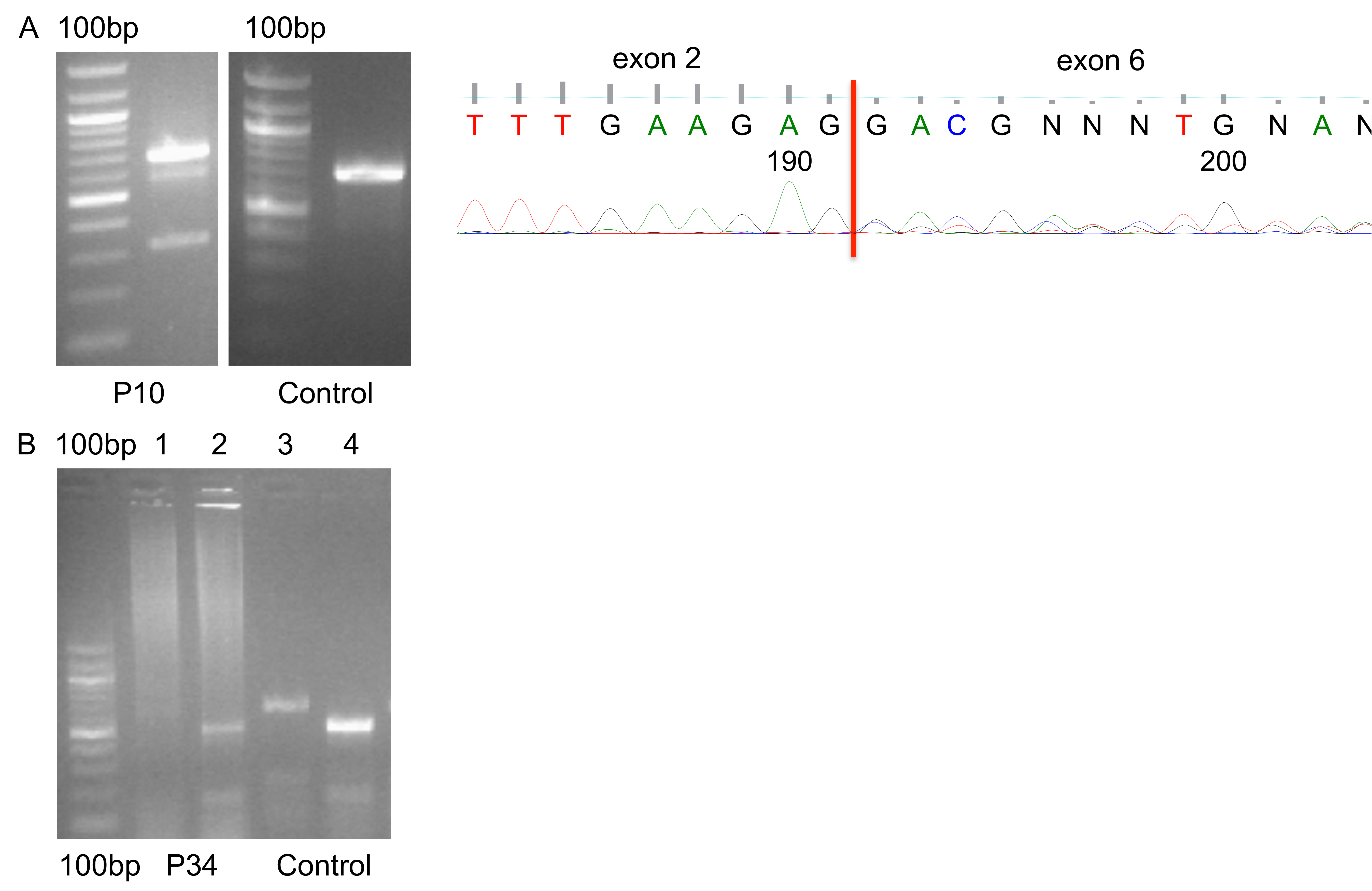

Supplement: S1 Fig — (A) Aberrant bands seen on agarose gel of RT-PCR product of TSC2 exons 1–7 for P10 are shown at left. At right is a Sanger sequencing trace demonstrating that the sequencing becomes duplicated at end of exon 2, and one of the adjacent sequences is that of exon 6. (B) Aberrant high molecular weight smear observed for P34 for RT-PCR amplicons covering exons 28–33 and 29–32 of TSC2 (lanes 1 and 2, respectively). The same amplicons for the control sample do not show this effect (lanes 3 and 4). The splice variant in P34 is located in TSC2 intron 29. RNA was prepared from puromycin-treated LCL cultures for both (A) and (B). (TIF) [file pgen.1005637.s001.tif]

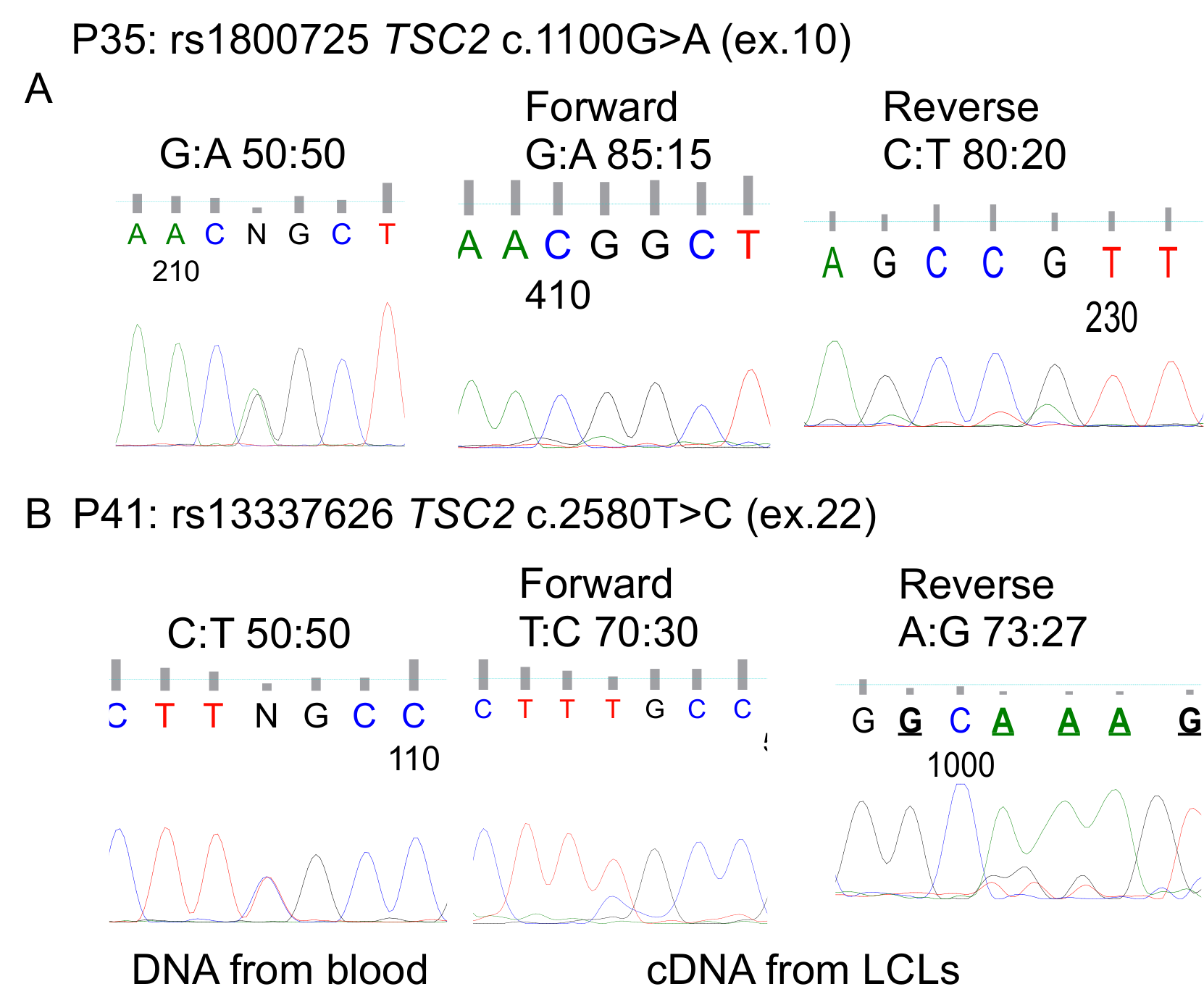

Supplement: S2 Fig — (A) P35, comparison of genomic DNA (left) and RT-PCR DNA (right, forward and reverse strand). Sanger sequencing demonstrates skewing of the allelic representation of the G and A alleles in the RT-PCR analysis. (B) P41, comparison of genomic DNA (left) and RT-PCR DNA (right, forward and reverse strand). Sanger sequencing demonstrates skewing of the allelic representation of the C and T alleles in the RT-PCR analysis. (TIF) [file pgen.1005637.s002.tif]

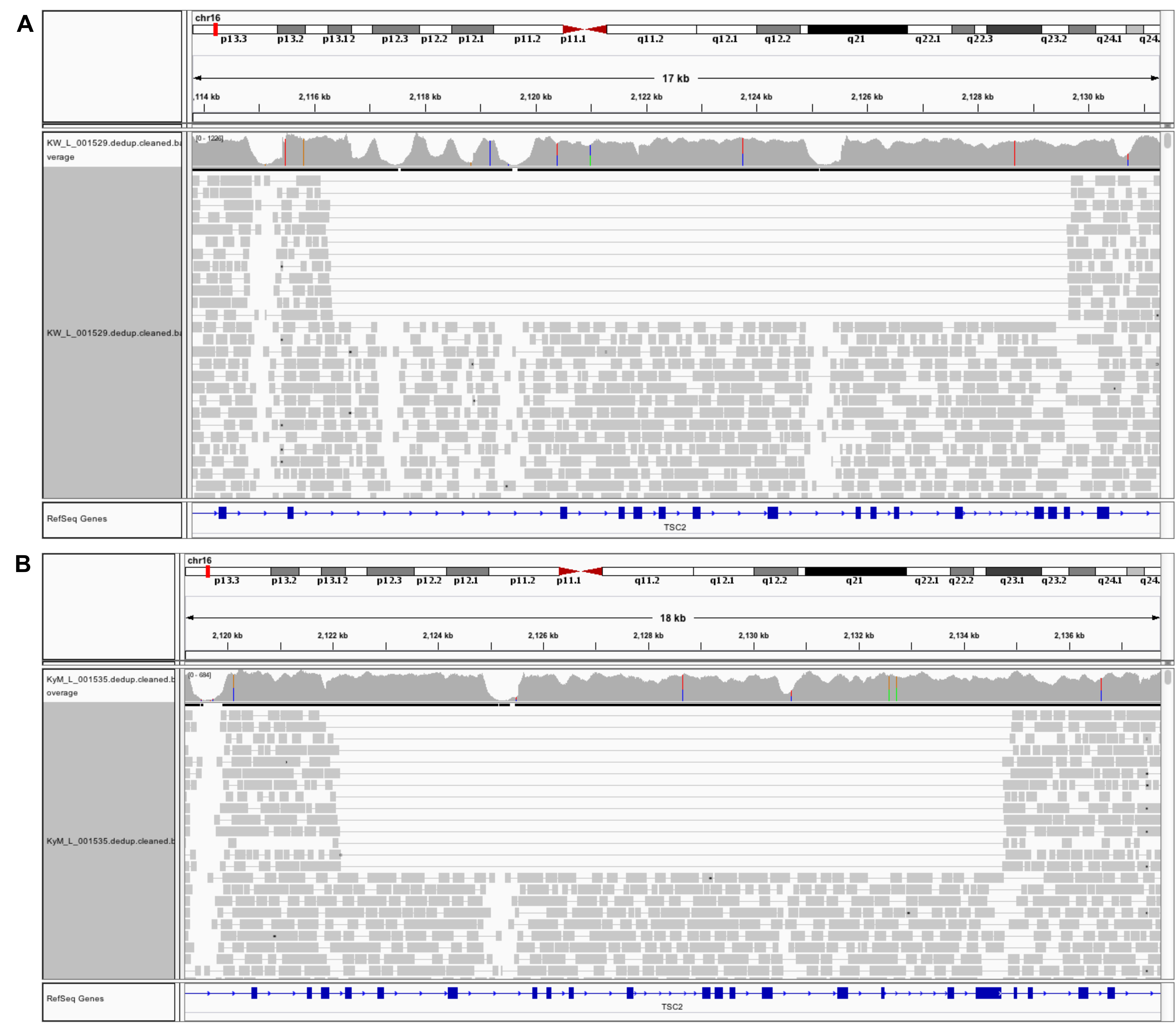

Supplement: S3 Fig — (A) 13,282 nt deletion seen in P9. (B) 12,591 nt deletion seen in P28. Paired end reads from NGS are displayed as boxes connected by a thin line, which represents the inferred insert size. (TIF) [file pgen.1005637.s003.tif]
