# Supplementary material for: Mosaic and Intronic Mutations in TSC1/TSC2 Explain the Majority of TSC Patients with No Mutation Identified by Conventional Testing
Source: PLoS Genet. 2015 Nov 5;11(11):e1005637. doi: 10.1371/journal.pgen.1005637 (PMC4634999; doi:10.1371/journal.pgen.1005637)
Supplement: S1 Table — (PDF) [file pgen.1005637.s004.pdf]

Table S1. Clinical features of 53 TSC NMI subjects

Patient	Age	Sex	Hypomelanotic macules (≥3)	Angiofibromas (≥3) or forehead plaque	Ungual fibromas (≥2)	Shagreen patch	Cortical tubers	Subependymal nodules	Subependymal giant cell astrocytoma	Retinal hamartomas	Seizures	Mood or anxiety disorder, ADHD	Autism, developmental delay	Cardiac rhabdomyoma	Angiomyolipomas (≥2)	Renal cysts	Lymphangioleiomyomatosis	Sporadic (S) or familial (F)
P1	5y	M	1	1	0	0	mult	mult	0	1	3	0	1	1	2	2	ND	S
P2	20y	M	1	1	0	1	mult	mult	0	1	3	1	0	1	0	0	ND	S
P3	31y	F	1	3	1	1	1	0	0	1	1	0	1	0	2	2	0	F
P4	9y	M	1	1	0	0	mult	mult	0	0	3	1	1	1	0	2	ND	S
P5	57y	F	0	2	1	0	0	0	0	0	0	1	0	0	2	1	0	F
P6	4y	M	1	0	0	0	1	1	0	0	2	0	1	1	0	0	ND	S
P7	11y	M	0	2	0	0	1	0	0	0	0	0	0	0	0	0	ND	S
P8	7y	F	1	1	0	1	1	1	0	0	0	0	0	0	1	1	ND	S
P9	1y	F	1	0	0	0	1	0	1	0	1	0	0	1	ND	ND	ND	S
P10	5y	F	1	0	0	1	0	1	0	0	2	1	1	0	0	1	ND	S
P11	8y	F	1	2	1	1	mult	mult	0	1	2	0	1	1	2	2	ND	S
P12	4y	M	0	2	0	0	1	1	0	0	1	0	1	0	0	0	ND	S
P13	14y	M	1	0	0	0	1	1	0	1	0	1	1	0	0	0	ND	S
P14	23y	M	0	2	0	0	0	1	0	0	0	0	0	0	2	0	ND	S
P15	40y	F	0	1	1	0	0	0	1	ND	0	0	0	0	2	2	1	S
P16	2y	M	1	0	0	0	1	0	1	0	0	0	0	1	0	0	ND	S
P17	2y	F	0	1	0	0	1	0	0	0	2	0	0	1	0	0	ND	S
P18	2y	F	1	0	0	0	0	mult	0	0	1	0	0	ND	0	1	ND	S
P19	33y	F	1	2	0	0	0	0	0	0	0	0	0	0	2	0	0	S
P20	30y	M	0	1	1	0	0	1	0	0	0	0	0	0	3	0	ND	S
P21	24y	M	1	2	1	1	mult	mult	1	1	3	0	0	1	2	1	ND	S
P22	fetus		ND	ND	ND	ND	ND	1	ND	ND	ND	ND	ND	1	ND	ND	ND	S
P23	9y	M	0	2	0	0	0	1	0	0	0	0	0	0	0	0	ND	S
P24	10y	M	1	0	0	1	0	1	0	0	0	0	0	0	0	0	ND	F
P25	19y	F	1	0	1	0	mult	1	0	0	0	1	0	0	0	1	ND	F
P26	6y	F	1	1	0	0	1	1	1	0	2	0	0	0	0	2	ND	S
P27	36y	M	0	1	1	0	mult	mult	0	1	1	0	0	ND	2	0	ND	S
P28	8y	F	1	2	0	1	mult	mult	0	1	2	1	1	0	1	1	ND	S
P29	26y	F	1	3	0	1	mult	1	1	0	0	0	0	1	0	0	0	S
P30	33y	M	0	1	0	1	0	0	0	0	0	0	0	0	2	0	ND	S
P31	18y	F	1	1	0	0	1	0	0	0	1	1	1	1	1	1	ND	S
P32	17y	F	1	1	0	1	1	0	0	1	1	0	1	1	0	1	ND	S
P33	8y	M	1	1	0	0	1	1	0	0	1	1	1	1	1	1	ND	S
P34	22y	F	1	1	0	1	mult	1	1	0	1	0	1	ND	3	2	0	S
P35	13y	M	1	2	0	0	1	0	0	0	2	0	2	1	0	0	ND	S
P36	30y	M	1	2	0	0	mult	1	0	0	2	1	1	0	0	1	ND	F
P37	7y	F	1	0	0	0	mult	mult	0	0	3	0	2	1	2	2	ND	S
P38	1y	M	1	0	0	0	1	1	0	0	3	0	0	0	0	0	ND	F
P39	18y	F	1	3	1	1	1	1	0	0	1	0	0	0	0	4	ND	S
P40	33y	M	1	2	1	1	mult	1	0	0	1	0	0	0	2	1	ND	S
P41	15y	F	0	1	0	1	mult	mult	1	1	3	0	1	0	0	0	ND	S
P42	25y	F	0	2	0	0	1	0	0	0	0	0	0	0	1	1	0	S
P43	27y	F	1	2	0	1	1	1	0	0	3	1	2	0	2	0	0	S
P44	20y	F	0	0	0	0	1	0	0	1	0	0	0	0	2	1	ND	S
P45	19y	F	1	2	0	0	0	0	0	ND	0	0	0	0	3	2	ND	S
P46	13y	F	1	3	0	1	mult	mult	0	0	3	1	1	1	2	2	ND	S
P47	1y	M	1	0	0	0	mult	1	0	1	2	0	0	0	0	3	ND	S
P48	4y	M	1	0	0	0	1	1	0	ND	2	1	0	0	0	0	ND	S
P49	33y	M	0	1	0	0	1	0	0	0	0	0	0	0	0	0	ND	S
P50	23y	F	0	0	0	0	1	0	0	0	1	0	0	0	2	0	0	S
P51	34y	F	0	2	0	0	0	1	0	0	0	0	0	ND	2	1	0	F
P52	1y	M	1	0	0	0	1	1	0	0	2	0	0	1	0	2	ND	S
P53	22y	M	1	1	1	0	mult	1	0	0	1	0	1	0	1	1	ND	S
			0 no 1 yes	0 no grades 1-3	0 no 1 yes	0 no 1 yes	0 no 1 yes	0 no 1 yes	0 no 1 yes	0 no 1 yes	0 never grades 1-3	0 no 1 yes	0 no 1 mild	0 no 1 yes	0 no grades 1-3	0 no grades 1-3	0 no 1 yes	

ND, not done or data not available

mult - multiple

grade 1: macular (flat) lesions only on cheek
grade 2: papular lesions, < 3 mm diameter
grade 3: papular lesions, > 3 mm diameter;

1 in the past
2 chronic
3 chronic + infantile spasms

2 moderate
3 severe

Renal angiomyolipoma
grade 1: one or more, all < 1 cm diameter
grade 2: multiple, one or more > 1 cm, and all < 4 cm in diameter
grade 3: multiple, one or more > 4 cm

Renal cysts
grade 1: 0 - 2 small (< 2 cm) cysts
grade 2: > 2 small (< 2 cm) cysts
grade 3: > 2 and at least one > 2 cm
grade 4: classic polycystic kidney disease- multiple cysts with renal enlargement
