# Supplementary material for: Mosaic and Intronic Mutations in TSC1/TSC2 Explain the Majority of TSC Patients with No Mutation Identified by Conventional Testing
Source: PLoS Genet. 2015 Nov 5;11(11):e1005637. doi: 10.1371/journal.pgen.1005637 (PMC4634999; doi:10.1371/journal.pgen.1005637)
Supplement: S2 Table — (PDF) [file pgen.1005637.s005.pdf]

Mutant allele frequency (AF) for mutations confirmed by repeat NGS

**Subject**	**Mutation**			**Mutation**			**Mutation**		
**P14**	*TSC2* c.976-15G>A			*TSC2* c.1372C>T; p.R458*			*TSC2* c.4490C>T; p.P1497L		
Sample	Reference reads	Mutant reads	Mutant AF	Reference reads	Mutant reads	Mutant AF	Reference reads	Mutant reads	Mutant AF
Angiofibroma	717	68	8.66%	251	13	4.92%	722	15	2.03%
Angiofibroma (repeated)	10695	1051	8.95%	5963	469	7.29%	78201	1001	1.26%
Blood	15920	43	0.27%	6937	8	0.11%	137931	172	0.12%
Saliva	9580	11	0.11%	2831	8	0.28%	52424	60	0.11%
Normal skin	4577	29	0.63%	17736	14	0.08%	149054	914	0.61%
Control average and range (n=25)	13705 (5628-29912)	22 (5-52)	0.16% (0.07-0.43%)	14330 (2156-28066)	19 (6-37)	0.13% (0.09-0.28%)	28827 (14673-59720)	20 (6-34)	0.07% (0.03-0.1%)

**P17**	*TSC2* c.2647C>T; p.Q883*		
Sample	Reference reads	Mutant reads	Mutant AF
Blood	1796	10	0.55%
Blood (repeated)	1159710	8097	0.69%
Angiofibroma	358167	2792	0.77%
Control average and range (n=25)	16428 (6365-35240)	17 (7-33)	0.11% (0.07-0.16%)

**P27**	*TSC2* c.2356_2362delCGCGAGA		
Sample	Reference reads	Mutant reads	Mutant AF
Blood	7648	202	2.57%
Blood (repeated)	97546	3009	2.99%
Ungual fibroma	19509	3251	14.28%
Angiofibroma	102567	7000	6.39%
Normal skin	40934	2222	5.15%
Control average and range (n=25)	20285 (8548-43325)	0 (0-1)	0% (0-0.01%)

**P30**	*TSC2* c.1413_1414delT*G*		
Sample	Reference reads	Mutant reads	Mutant AF
Blood	11641	611	4.99%
Blood (repeated)	50053	2798	5.29%
Normal skin cell culture	225716	226	0.10%
Control average and range (n=25)	15718 (2316-30572)	0 (0-1)	0% (0-0.01%)

**P32**	*TSC2* c.4850-1G>A		
Sample	Reference reads	Mutant reads	Mutant AF
Blood	5704	41	0.71%
Blood (repeated)	952673	2663	0.28%
Control average and range (n=25)	5717 (5238-5877)	5 (1-11)	0.09% (0.02-0.20%)

**P42**	*TSC2* c.4530_4537delTGGCGACG			*TSC2* c.1769T>G; p.L590R		
Sample	Reference reads	Mutant reads	Mutant AF	Reference reads	Mutant reads	Mutant AF
Angiofibroma1	752	36	4.57%	801	22	2.67%
Angiofibroma1 (repeated)	24963	593	2.32%	94967	1595	1.65%
Angiofibroma2	14758	128	0.86%	21995	2	0.01%
Angiofibroma3	56089	625	1.10%	65701	14	0.02%
Normal skin	41923	153	0.36%	70348	7	0.01%
Blood	16454	0	0%	31173	5	0.02%
Saliva	14576	3	0.02%	28339	5	0.02%
Control average and range (n=25)	5443 (2864-5705)	0	0%	20072 (8616-36590)	8 (2-16)	0.04% (0.01-0.07%)

**P49**	*TSC2* c.848+281C>T			*TSC2* c.4619delA		
Sample	Reference reads	Mutant reads	Mutant AF	Reference reads	Mutant reads	Mutant AF
Angiofibroma	640	90	12.33%	651	24	3.56%
Angiofibroma (repeated)	2585	342	11.68%	96586	2762	2.78%
Blood	107402	8000	6.93%	552649	0	0%
Semen	14077	36	0.25%	82037	0	0%
Control average and range (n=25)	13162 (6233-33397)	18 (7-40)	0.14% (0.08-0.24%)	14776 (7261-30674)	0	0%

**P51**	*TSC2* c.4180_81delCT		
Sample	Reference reads	Mutant reads	Mutant AF
Blood	128434	91	0.07%
Saliva	53055	113	0.21%
Angiofibroma 1	46902	464	0.98%
Angiofibroma 2	467659	6445	1.36%
Daughter's blood	19731	15479	43.96%
Control average and range (n=25)	16363 (8400-34198)	1 (0-5)	0.005% (0-0.03%)

**P15**	*TSC2* c.4238insGGTTAAG		
Sample	Reference reads	Mutant reads	Mutant AF
Blood	1209	31	2.50%
Blood (repeated)	3378	113	3.24%
Control average and range (n=25)	15856 (7951-33181)	0	0%

**P20**	*TSC2* c.5228G>A; p.R1743Q		
Sample	Reference reads	Mutant reads	Mutant AF
Blood	4740	69	1.43%
Blood (repeated)	677	9	1.31%
Control average and range (n=25)	11045 (5633-23014)	12 (1-30)	0.1% (0.01-0.17%)

**P31**	*TSC2* c.1831C>T; pR611W		
Sample	Reference reads	Mutant reads	Mutant AF
Saliva	5812	70	1.19%
Saliva (repeated)	317637	3133	0.98%
Control average and range (n=25)	5800 (4030-5944)	6 (0-11)	0.09% (0-0.18%)

**P45**	*TSC2* c.4051G>T; p.E1351*		
Sample	Reference reads	Mutant reads	Mutant AF
Saliva	2912	317	9.82%
Saliva (repeated)	123539	5728	4.43%
Control average and range (n=25)	13958 (7102-29216)	5 (1-12)	0.03% (0.01-0.12%)
