# Supplementary material for: Mosaic and Intronic Mutations in TSC1/TSC2 Explain the Majority of TSC Patients with No Mutation Identified by Conventional Testing
Source: PLoS Genet. 2015 Nov 5;11(11):e1005637. doi: 10.1371/journal.pgen.1005637 (PMC4634999; doi:10.1371/journal.pgen.1005637)
Supplement: S3 Table — (PDF) [file pgen.1005637.s006.pdf]

Table S3. Oligonucleotide primers used in the study.

Gene	PCR fragment	Forward 5'-3'	Reverse 5'-3'
Long-range PCR			
TSC1	promoter-intron1	cgggcttctgaaaatgtttagcc	ggctcttcatcctccagttcctc
TSC1	intron1-intron2	tcttaggctcaggaatggggagcat	caccacacctggccaaaaatgtctt
TSC1	intron2-intron8	acctccctgtagccagtggtatttgggtgtgcaa	aggcacttgtgctgcaactttctccactctgct
TSC1	intron8-intron12	acttgggctggcatggtcccgcttgttctttgct	accacccaggggtcggcagatcacaccttgaga
TSC1	intron12-intron20	ttgagaatcactgcactcggctgac	tctcccctttctgttcccagtcat
TSC1	intron20-intron22	tgtagcctcacctgcgctattgtcaggagggggaga	gggggttagggcgggtggaggggaaggt
TSC1	intron22-beyond 3'UTR	gtgggctagcggagttcagtgtcag	tcacccaaggtagccaaaccagacc
TSC2	promoter-exon1	cagcaggtcctcaacggctgc	ggtggaccaggacgcaccaga
TSC2	exon1-intron6	gctgtagttgagttctcccagggagtg	gactcctgaggctcagagagaccgag
TSC2	intron6-intron11	cttggagagagggtgccatg	aatcagacctgtctccggtg
TSC2	intron11-intron16	ctgagggtgtctccatgcg	ccacaagagcaggaggaagg
TSC2	intron16-intron26	ggttgggttttactttttgctgctgtg	atgcaacctttccacccctcgtc
TSC2	intron26-intron33	caggaggccgtaacctagtg	cgagggatgtggaagaacat
TSC2	intron33-3'UTR	tcaggggatgctgatacctc	accacttctgtgggcatgg
PCR to confirm NGS findings by Sanger sequencing			
TSC1	intron6-intron8	gtcctgctggcagccacttgt	tgagtgcttccaagtggactgattct
TSC1	intron8-intron9	cccgcttgttctttgctatc	ggagggacatgcaaatttaaga
TSC1	intron14-intron15	caagtgatcctccctcctca	agagaaccagctgcctcaag
TSC1	intron15-intron17	tgcagattttgaccacaagg	tgctgacccaaaacaaaaca
TSC1	intron20-intron22	tgccttctcagtccttcttaca	gtgggtctctgacacggagt
TSC2	intron2-intron3	ggagatacgagctttggaggt	cagacctcatgacaccagga
TSC2	intron3-intron4	tgcagacctgtctcttgcag	tccttcccatccaggttaca
TSC2	intron7-intron8	agaaccctgctgcctctgt	ctagtggcagcctctggaac
TSC2	intron8-intron9	cccaagcacagggacctc	gcctggaaaggcctagaaat
TSC2	intron9-intron10	cccctgataaacgtgtggtg	acacggttctggcagtctct
TSC2	intron12-intron13	gagctctgtgccctgtgtg	accgaagtcccaggcagag
TSC2	intron15-intron16	gggtttgaaggtcgtgtgtt	ctgccgagacggggatac
TSC2	intron19	ccaagggcatgtcactgaatg	caggaaagggggcaaaggg
TSC2	intron28-intron29	gtggtggtcaccagtcctc	cgcccctgagtgtccacagc
TSC2	intron29-intron30	ggctccgacatcgtgg	acctcccacgggtcagcctc
TSC2	intron33-intron35	cctagggctggctggaac	gccctgtccaggcaccta
TSC2	intron37-intron40	ggtgtctagcagtgcaacca	gctgagggagccccatatt
PCR for short amplicon NGS			
TSC2	intron8	catgctcggacgtcctcc	ggtgagggcaagggcaac
TSC2	intron9-exon10	agcaagcaagcagctctgac	ggtgatggacaggacgatct
TSC2	intron12-exon13	gctggtgtggggctgt	cacgtccagcaccttgatg
TSC2	intron15-exon16	gtgagctgcgtcctctctct	tgtgcttgtagtggagctgaa
TSC2	exon16-intron16	acgcgtgtgtatgagatgct	aaccccagccaccatacct
TSC2	intron20-exon21	ggcctgaggtgtcctgtct	agatggacaaggccacga
TSC2	intron22-exon23	catgccctggggatgtttcc	tatgacgtgatgggccagac
TSC2	intron32-exon33	gggatggtcctttctagtcg	aggagacgactcgctcgat
TSC2	exon33	gggaccctggggacaa	cgaccaggcagcactttc
TSC2	exon33	tgagcaagtccagctcctct	gccttaacctcagggctca
TSC2	exon33-intron33	agcctccaatgcagagaaag	gcaagtgaggcaccaggtt
TSC2	intron33-exon34	ccctgtgcagtttcgtgtt	ctacctcattgggcagcag
TSC2	exon35	tcacagtcctttgagcggtc	cacctggccttctccaacat
TSC2	intron36-exon37	cagcactggccccacaaa	tgcttgtccacgtccttgg
RT-PCR			
TSC2	exon1-exon7	gccaaaccaacaagcaaaga	caggcagttgtagcagacca
TSC2	exon12-exon18	acggctggattcagaacct	cggaagagcagggagtagg
TSC2	exon18-exon23	ccctttctcctcccacagg	cctgatgaaccacatggcta
TSC2	exon28-exon33	gcatgtgagacagaccaagg	aggacgacctgctgtaggc
TSC2	exon29-exon32	catggtcttcgagttggcg	tcaacgtcctccaaccct
